# A stable, engineered TL1A ligand co-stimulates T cells via specific binding to DR3

**DOI:** 10.1038/s41598-022-24984-y

**Published:** 2022-11-29

**Authors:** Adam Zwolak, Szeman Ruby Chan, Paul Harvilla, Sally Mahady, Anthony A. Armstrong, Leopoldo Luistro, Ninkka Tamot, Douglas Yamada, Mehabaw Derebe, Steven Pomerantz, Mark Chiu, Rajkumar Ganesan, Partha Chowdhury

**Affiliations:** 1 Biologics Discovery, Janssen Research & Development, LLC, Spring House, PA 19477 USA; 2 Oncology Discovery, Janssen Research & Development, LLC, Spring House, PA 19477 USA; 3 Merck Research Laboratories, Discovery Biologics, Protein Sciences, South San Francisco, CA USA; 4 Tavotek Biotherapeutics, Spring House, PA USA; 5 Immunotherapeutics, Amgen, South San Francisco, CA USA; 6 Cell Engineering and Early Development, Janssen Research & Development, Spring House, PA USA

**Keywords:** Tumour-necrosis factors, Molecular medicine, Cytokines

## Abstract

TL1A (TNFSF15) is a TNF superfamily ligand which can bind the TNFRSF member death receptor 3 (DR3) on T cells and the soluble decoy receptor DcR3. Engagement of DR3 on CD4+ or CD8+ effector T cells by TL1A induces downstream signaling, leading to proliferation and an increase in secretion of inflammatory cytokines. We designed a stable recombinant TL1A molecule that (1) displays high monodispersity and stability, (2) displays the ability to activate T cells in vitro and in vivo, and (3) lacks binding to DcR3 while retaining functional activity via DR3. Together these results suggest the TL1A ligand can be amenable to therapeutic development on its own or paired with a tumor-targeting moiety.

## Introduction

Cytotoxic (CD8+) T cells are part of a potent adaptive immune system to eliminate tumor cells by recognition of tumor peptides presented on major histocompatibility (MHC) class I molecules on tumor cells via their T cell receptors (TCRs). However, tumor infiltrating lymphocytes (TILs) can fail to suppress tumor growth due to a variety of immunosuppressive mechanisms exploited by tumor cells. Three hallmarks of T cell exhaustion are upregulation of immune checkpoint receptors, lower inflammatory cytokine secretion, and altered gene expression profiles^1^. The best characterized immune checkpoint modulatory axis is that of programmed death 1: ligand (PD-1: PD-L1)^2^. Indeed, antibodies that block the PD-1: PD-L1 interaction have shown marked clinical success in subsets of patients highlighting both the promise of immune checkpoint blockade therapy and the need for therapeutic molecules which can modulate other immune checkpoint axes^3^.

T cell activation occurs via a two-signal mechanism^4^. First, the T cell receptor (TCR) complex recognizes its cognate peptide MHC complex. Second, a host of co-signaling receptors determine whether the T cell becomes activated or suppressed. The co-stimulatory receptor CD28 co-localizes with the TCR to the central super-molecular activating center (cSMAC) while other immunomodulatory receptors may localize to the peripheral or distal super-molecular activating centers (pSMAC or dSMAC) to guide signaling for T cell activation or differentiation into regulatory T cells (Tregs)^5^.

One co-stimulatory receptor, TNF-like factor 1A (TL1A) protein belongs to the TNF family and is expressed on a variety of cell types as a type II single-pass transmembrane protein which can be cleaved from the cell surface. Soluble or membrane-bound TL1A binds to the co-stimulatory death receptor 3 (DR3) on T cells or NK cells^6^. Additionally, TL1A can also bind to the soluble decoy receptor DcR3 (TNFRSF6B) at higher affinity than to DR3 thereby preventing TL1A from stimulating T cells^7,8^. DcR3 is structurally homologous to DR3 but shares only ~ 20% sequence identity with DR3. DcR3 can therefore serve as a sink to prevent TL1A from activating T cells. DcR3 is up-regulated in a variety of tumors and is associated with metastasis^7–10^. DcR3 displays promiscuity in its abilities to bind to three TNFSF ligands: TL1A, Fas ligand (FasL), and LIGHT (homologous to *l*ymphotoxin, exhibits inducible expression, and competes with HSV *g*lycoprotein D for *h*erpesvirus entry mediator (HVEM), a receptor expressed on T cells). These interactions may sequester TL1A from engaging DR3 thereby down-regulating cell immune response to tumors^11^.

TL1A blocking antibodies have been used in the clinic to treat ulcerative colitis and other inflammatory conditions^12^. Interaction between TL1A and DR3 contributes to T cell activation, leading to increased inflammatory cytokine production and T cell proliferation. Thus, the potential of employing such interaction to overcome T cell exhaustion in solid tumors makes DR3 an attractive immune checkpoint target. Nonetheless, significant challenges to therapeutic targeting of the TL1A:DR3 axis remain. The 3:3 stoichiometry of trimeric TL1A and DR3 binding is finely tuned suggesting that a ligand-based approach may be more amenable to T cell activation compared to bivalent antibody-based approaches. However, the high levels of DcR3 require either higher dosing regimens or engineering to eliminate this interaction as a sink in tumor micro-environment.

Here, we sought to engineer a recombinant TL1A ligand that would be suitable for therapeutic development for T cell activation. The desired engineered TL1A therapeutic would satisfy 3 criteria: (1) specific binding to DR3 but not DcR3; (2) display a long serum half-life; and (3) demonstrate structural stability. TL1A ligand has been engineered to modulate affinity for DcR3^13^. Additionally, the crystal structure of TL1A bound to DcR3 showed the molecular basis for these interactions^14^. The close structural homology but low sequence identity of DR3 and DcR3 suggest that selective mutations could influence specific binding to DR3 over DcR3.

We tested several molecular architectures of TL1A, including single-chain (sc) variants, in which three TL1A monomers were fused into a single polypeptide. TL1A ligands were tested as fusion proteins fused to either human serum albumin (HSA) or to an Fc domain, both of which have been demonstrated to increase the serum half-life of biologics^15^. We determined the minimal sufficient fragment of TL1A suitable to form a stable trimer capable of binding DR3. A structure and model-guided strategy was used to design and engineer TL1A mutations, leading to a molecule that retained DR3 binding with affinity similar to wild-type TL1A but displayed no measurable binding to DcR3. This Fc-TL1A fusion displayed T cell co-stimulation in in vitro and in vivo models.

## Results

### Fc-fused recombinant TL1A molecules display homogeneity and binding activity

**Figure 1:**
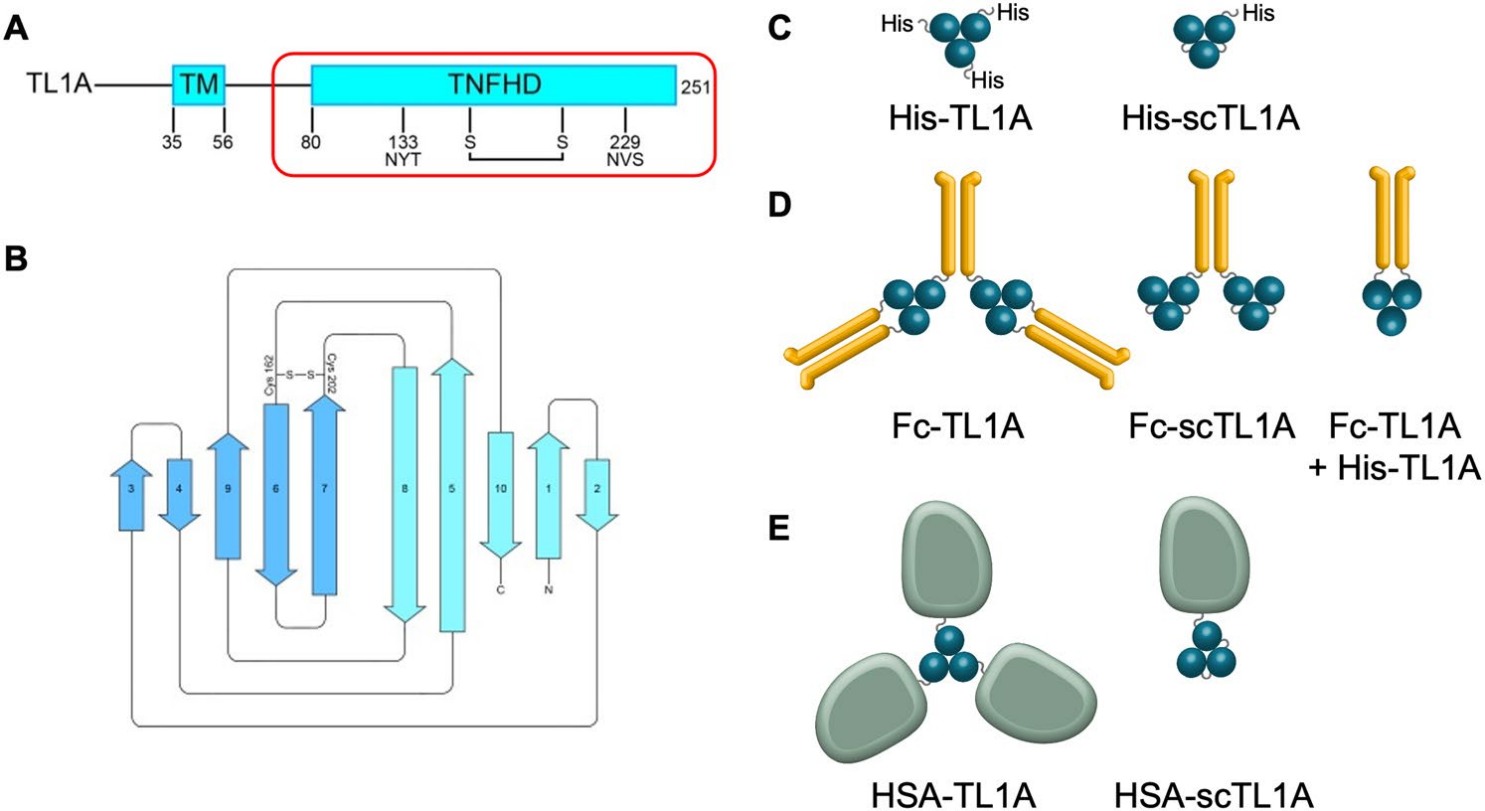
Several Architectures of TL1A Ligand were Evaluated. (**A**) TL1A is a type I transmembrane (TM) protein. Ligand designs focused on the C-terminal TNF homology domain (TNFHD), which contributes to binding to DR3 and DcR3. The positions of two N-linked glycosylation sites and disulfide bonds are indicated. (**B**) Secondary structure of the TNF homology jellyroll domain, which features ten anti-parallel b-strands which fold into a b-sandwich structure. Ligand designs fell into three categories: (**C**) His-TL1A or His-scTL1A, (**D**) Fc-TL1A, Fc-scTL1A, or Fc-His-TL1A, and (E) HSA-TL1A or HSA-scTL1A. Note that the Fc + His-TL1A molecule was generated by co-transfection of His-TL1A with Fc-TL1A at a molar DNA ratio of 1:2, and the desired product was purified by protein A and Ni–NTA tandem affinity chromatography. Images were generated with Adobe Illustrator (Version 25): https://www.adobe.com/products/illustrator.html.

**Figure 2:**
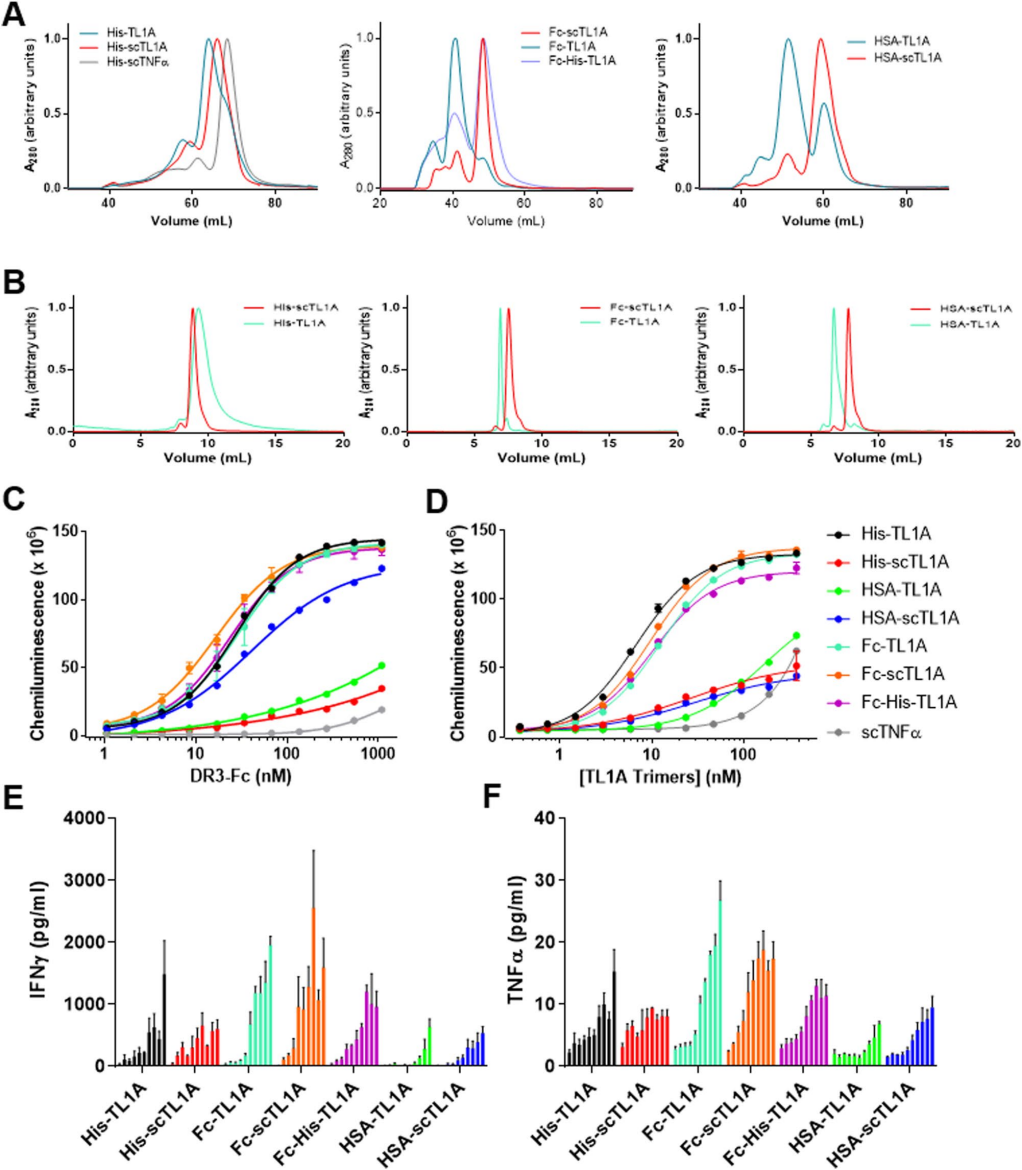
Fc-scTL1A Architecture Displayed High Monodispersity and Functional Activity. (**A**) Preparative gel-filtration analysis of TL1A constructs (indicated in legend). Recombinant TNFa was used as a control to show the population of trimer vs. oligomers. Main peaks in each chromatogram represent the target oligomeric species indicated in the main text. Off-target high molecular weight (HMW) oligomers and off-target low molecular weight (LMW) species are indicated in the chromatograms. (**B**) Analytical SEC analysis of TL1A constructs after preparative gel-filtration purification to isolate desired oligomeric species. The purified species were used in functional assays. (**C**, **D**) ELISA analysis of the ability of TL1A constructs to bind DR3 (left) or DcR3 (right). Molar concentrations of TL1A for each construct are normalized to concentration of TL1A trimers in each molecule. (**E**, **F**) Pan T cells from healthy donors were incubated with plate-bound anti-CD3 Ab (0.01 mg/mL). TL1A ligands at 0, 0.03, 0.1, 0.3, 1, 3, 10, 30 or 100 nM (trimer molarity) were added to aCD3-activated T cells. Levels of IFNg (left) and TNFa (right) produced by the activated T cells are shown. Stastical significance was determined using an unpaired T-test of unstimulated vs stimulated cells for each construct. Results represent averages from three independent experiments. Graphs were generated using Graphpad Prism (Version 9): https://www.graphpad.com/scientific-software/prism/.

Since TL1A is a type II transmembrane protein, and we focused on the C-terminal extracellular domain comprising the TNF homology domain which forms into a homotrimer of jellyroll fold-based subunits and binds DR3 / DcR3 (Fig. 1A,B). The jellyroll fold comprises two sets of five b-sheets which are connected by unstructured loops. Since soluble TL1A (Fig. 1C) has a short serum half-life, TL1A ligands were fused to the C-terminus of either an IgG1 Fc or to human serum albumin (HSA) due to the ability of HAS to extend serum half-life (Fig. 1D,E, respectively). TL1A ligands were designed to trimerize either through native, non-covalent interactions or as a single-chain (sc) using a linker peptide (Fig. 1C–E). Because the N- and C-termini of the TL1A jellyroll domains are close in the structure, and we designed the TL1A ligand to contain residues 84-251 fused by a GGSGGSGGS linker which was sufficient to maintain full activity compared to the non-fused molecule. The quaternary structure of the Fc fusion had a 3:2 ratio of Fc:TL1A trimers; the Fc-scTL1A fusion had a 1:2 ratio of Fc:scTL1A since the linker in the single-chain molecule formed intra-polypeptide trimers; and the combination of Fc-TL1A + TL1A His-tag monomer had a 1:1 ratio of Fc:TL1A (Fig. 1D). For HSA-TL1A, the ratio of HSA to TL1A was 3:1, and for HSA-scTL1A, the ratio was 1:1 (Fig. 1E). We evaluated each format for its expression level, monodispersity, binding activity, and ability to activate T cells. Since several TNFSF ligands have been developed for clinical studies or as therapeutics^1^, we used a scTNFα molecule as a comparison for protein monodispersity. After expression and affinity capture, proteins were purified by preparative gel filtration, and the relative population of target species of each molecule was quantified (Fig. 2A, Table 1). Interestingly, all constructs in which the TL1A subunits were not fused with a linker peptide displayed off-target low molecular weight (LMW) species, suggesting that TL1A subunits could dissociate in solution. This was surprising since the subunit interface is comprised mainly of hydrophobic residues and comprises > 4000 Å^2^ (based on PDB ID 2RE9). When comparing the scTL1A constructs; His-scTL1A, Fc-scTL1A, and HSA-scTL1A; each was > 70% target species with Fc- and HSA-scTL1A displaying ~ 80% target oligomer. We further assessed the molecular weights of each species of the Fc-scTL1A by size-exclusion chromatography multi-angle light scattering (SEC-MALS) (Supplementary Fig. S1A). The main species showed a molecular mass of 197 kDa, consistent with a single Fc-scTL1A homodimer while the early eluting peak showed a molecular mass of 447 kDa, consistent with a dimer of homodimers. The later eluting shoulder showed a molecular mass of 130 kDa and may have represented a half-Fc-scTL1A species. All TL1A constructs were purified by gel-filtration to > 95% target oligomer for binding analysis and functional testing (Fig. 2B).

**Table 1:** Measurement of Monodispersity of TL1A molecules.

Molecule	Target oligomer	% HMW* species	% Target species	% LMW* species
His-TL1A (TL1W2)	Trimer	22	54	24
His-scTL1A TL1W19	Monomer	27	73	NA
His-scTNFa	Monomer	30	70	NA
Fc-TL1A (TL1W3)	Hexamer	23	67	10
Fc-His-TL1A (TL1W61)	Dimer	46	46	8
Fc-scTL1A (TL1W14)	Dimer	22	78	NA
HSA-TL1A (TL1W9)	Trimer	10	60	30
HSA-scTL1A (TL1W15)	Monomer	18	82	NA

We tested whether each TL1A molecule could bind recombinant DR3 by ELISA (Fig. 2C,D, Table 2, Supplementary Fig. S1B-C). Molar concentrations of TL1A constructs were normalized to molarity of TL1A trimer, since the trimeric form is the functional unit for DR3 binding. This allowed comparison of the binding activity *per* TL1A trimer in each format, and therefore assessment of whether molecules having two TL1A trimers (Fc-TL1A or Fc-scTL1A) would display an increase binding avidity compared to molecules having a single TL1A trimer (His-TL1A, His-scTL1A, Fc-TL1A + His-TL1A, HSA-TL1A and HSA-scTL1A) (Fig. 1C,D,E). Most TL1A constructs displayed an EC_50_ for binding to immobilized DR3 ~ 26 nM. We also confirmed TL1A molecules could bind DR3 in the reversed format, where the TL1A molecules were immobilized and DR3 was titrated (Fig. 2D, Table 2, Supplementary Fig. S1). In this format, the molecules displayed EC_50_ ~ 3–7-fold tighter for binding, but the HSA-scTL1A construct failed to bind significantly, and thus, we relied on the immobilized TL1A for ELISA analysis. The two HSA-TL1A proteins displayed significantly weaker binding in both formats, suggesting that the HSA-fusion partner may have inhibited the ability of the TL1A moiety to bind its receptor (Fig. 2D). Additionally, the His-scTL1A molecule had an EC_50_ ~ 3-fold weaker than other molecules. As a negative control, scTNFα displayed no significant binding to DR3.

**Table 2:** ELISA results for TL1A constructs binding to DR3.

Molecule	Target oligomer	EC50 (nM): DR3 immobilized	EC50 (nM): TL1A immobilized
His-TL1A (TL1W2)	Trimer	6.8 ± 1.0	2.5 ± 1.0
His-scTL1A TL1W19	Monomer	27.3 ± 1.3	N.B
His-scTNFa	Monomer	N.B.*	N.B
Fc-TL1A (TL1W3)	Hexamer	12.4 ± 1.0	2.6 ± 1.1
Fc-His-TL1A (TL1W61)	Dimer	10.2 ± 1.0	2.2 ± 1.1
Fc-scTL1A (TL1W14)	Dimer	9.7 ± 1.0	1.5 ± 1.1
HSA-TL1A (TL1W9)	Trimer	167.6 ± 1.1	> 4,000 ± 283
HSA-scTL1A (TL1W15)	Monomer	29.2 ± 1.2	3.8 ± 1.1

*No binding.

### Recombinant TL1A molecules display costimulatory function

We next determined whether the TL1A constructs could co-stimulate T cells, leading to cytokine production using a T cell activation assay. In the absence of aCD3 antibody, the TL1A molecules could not stimulate T cells, as expected due to the lack of TCR stimulation (Fig. 2E,F). In the presence of sub-optimal aCD3 antibody concentrations (0.01 or 0.1 mg/mL), T cells could be stimulated by the TL1A molecules to produce IFNγ and TNFα in a dose-dependent manner. The HSA-TL1A molecules, which displayed only weak binding to DR3, likewise showed the weakest co-stimulatory activities compared to the other TL1A constructs. While the His-TL1A molecules displayed some co-stimulatory activity, the Fc-TL1A molecules displayed the highest abilities to co-stimulate T cells to produce cytokines. While all three Fc-TL1A formats could co-stimulate T cells, the Fc-scTL1A displayed the most consistent T cell co-stimulatory function (*p* < 0.0001 compared to unstimulated cells) and favorable monodispersity.

**Figure 3:**
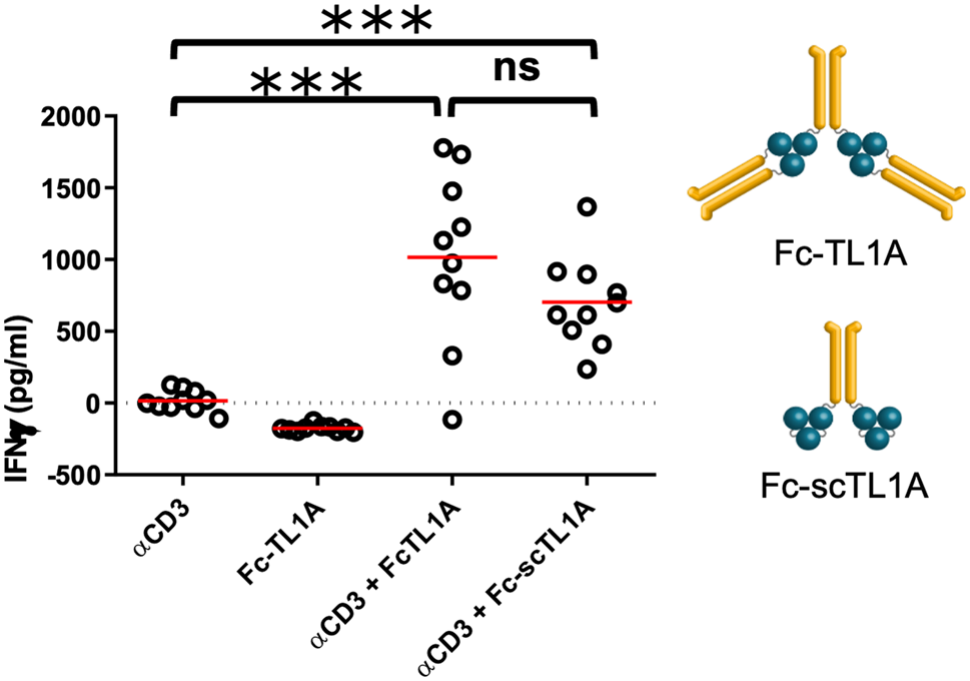
Fc-TL1A ligands activate T cells in vivo*.* Mice were treated with anti-CD3 antibody and Fc-TL1A or Fc-scTL1A, and serum concentrations of IFNg were measured. IFNg levels are shown in pg/mL and constructs are indicated on the graph. Stastical significance was determined using an unpaired T-test for each construct using Graphpad Prism (Version 9): https://www.graphpad.com/scientific-software/prism/.

To assess whether TL1A molecules could also co-stimulate T cells in vivo, we exploited the fact that human TL1A could cross-react with mouse DR3 (data not shown)^16^. C57BL/6 mice were injected with equal molarity of Fc-TL1A, Fc-scTL1A or Fc-His-scTL1A intraperitoneally, and anti-CD3 Ab intravenously (Fig. 3). Plasma samples were collected 6 h post-injection and assayed for IFNg levels. Treatment with either anti-CD3 antibody or Fc-TL1A alone had no effect on T cell activation, whereas treatment with anti-CD3 antibody in combination with either Fc-TL1A or Fc-scTL1A resulted in similar levels of T cell activation 1050 or 750 pg/mL, respectively), as determined by elevated levels of serum IFNg.

### Fc-scTL1A ligands could be optimized for monodispersity

**Figure 4:**
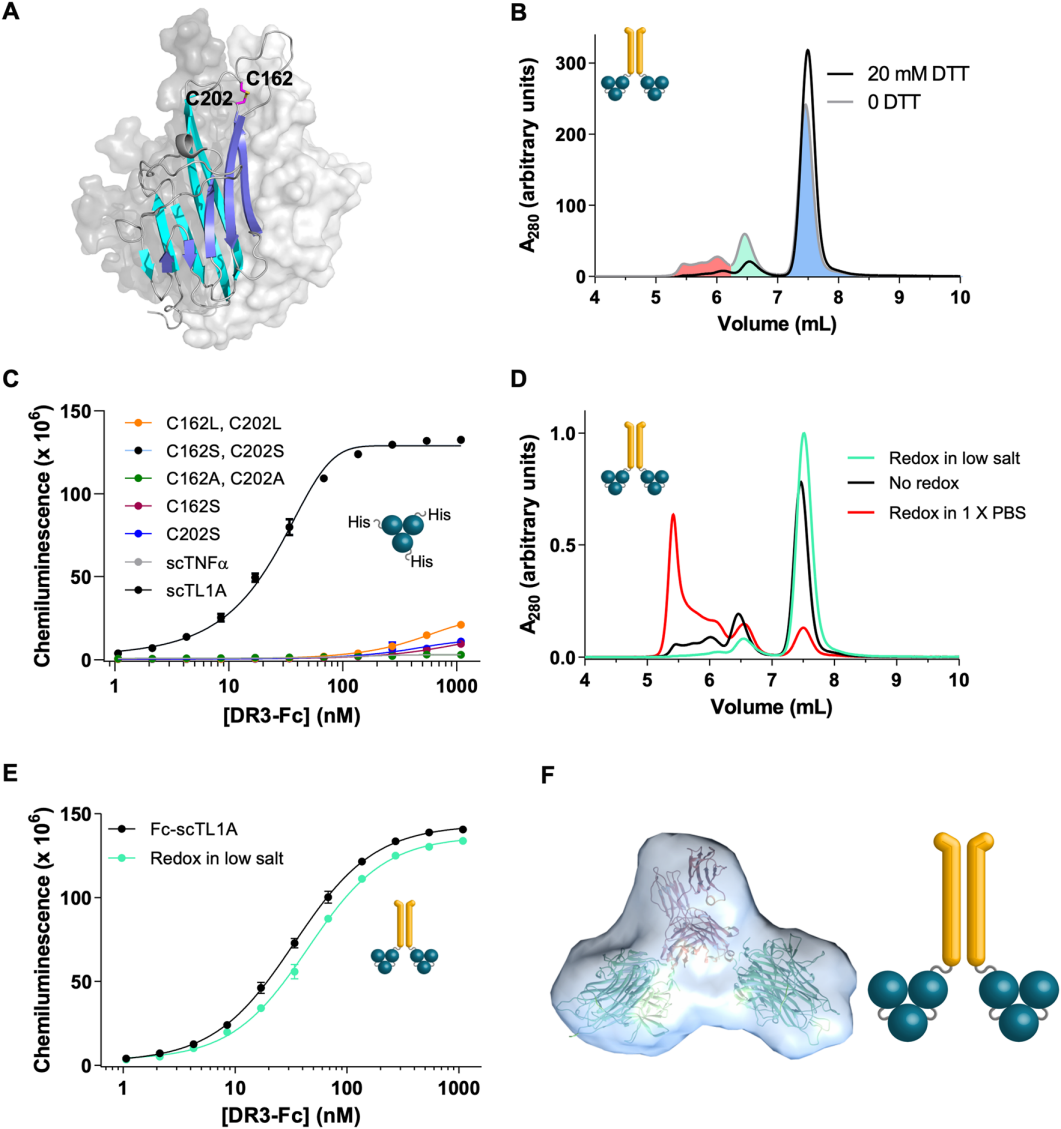
Fc-scTL1A was Optimized for Monodispersity. (**A**) Structural depiction of the crystal structure of the TL1A trimer, adapted from PDB ID 2RE9, showing the three subunits of TL1A. One subunit is shown in blue ribbons while the other two subunits are shown as gray surfaces. The position of the C162-C202 disulfide bond, which is critical for maintenance of DR3 binding is highlighted pink. (**B**) Analytical SEC analysis of Fc-scTL1A after protein A capture (gray line) showing target dimer species (blue fill), undesired tetrameric species (green fill), and undesired heterogeneous oligomeric species (red fill). Elution profile of Fc-scTL1A under reducing conditions is shown as the black trace. (**C**) ELISA analysis of the abilities of C162 / C202 mutants to bind DR3. TL1A variants were designed as His-TL1A (non-covalent trimers), and identities are indicated in the graph. (**D**) Analytical SEC analysis of Fc-scTL1A after protein A purification in non-reducing buffer (black trace), after redox in 1 × PBS (red trace) and after redox in low salt buffer (green trace). (**E**) ELISA comparison of the binding of Fc-TL1A purified by gel filtration (black) or after redox in low salt buffer to recover dimeric target species (green). (**F**) Solution X-ray scattering analysis of the Fc-scTL1A molecule showing the scTL1A moieties oriented away from the Fc. Manual docking of TL1A and Fc components of TL1A W14 into bead model for 11.85 mg/mL concentration. Fc dimer generated from PDB ID 1L6X. TL1a trimers are from PDB ID 2RE9. Structural images were generated using Pymol (Schrödinger): https://pymol.org/2/.

Although the Fc-scTL1A molecule displayed the most favorable properties for therapeutic development, the recombinant protein was ~ 20% oligomer after initial protein A-based affinity capture, and we asked whether the monodispersity of the molecule could be optimized. Although manufacturing-scale purification of biologics can apply ion-exchange-based methods to remove off-target species, this represents a significant cost-of-goods challenge and methods to either prevent aggregation during expression or recover this material as target species are desirable. Some TNFSF ligands contain two cysteine residues that form an intra-subunit disulfide bond between the membrane-distal CD and EF loops, but which are solvent exposed and may be reactive in solution (Fig. 4A). We asked whether the high molecular weight species were mediated by improper inter-molecular disulfide bonding by these cysteine residues using analytical size-exclusion chromatography (SEC) (Fig. 4B). Under reducing conditions (20 mM DTT), the population of high molecular weight species in the Fc-scTL1A molecule decreased from 22% to less than 10%, suggesting that improper inter-molecular disulfide bonding was largely responsible for the high molecular weight species. Surprisingly, mutation of C162, C202 or both cysteines to serine, leucine, or alanine resulted in loss of binding to DR3 (Fig. 4C). A previous study showed that the double mutant C162S, C202S had no impact on binding to DcR3^13^, and our ELISA and SPR-based binding analysis confirmed that the C162S, C202S TL1A variant could bind only DcR3 but not DR3 (Supplementary Fig. S2). This data suggested that either the cysteine residues themselves were involved in binding DR3 but not DcR3 or that the disulfide bond was necessary for a structural conformation required for binding DR3 only. Since these two cysteine residues were required for binding DR3, we asked whether formation of the high-molecular weight species could be prevented by a redox approach during purification. Incubation of Fc-scTL1A with 20 mM DTT followed by dialysis into 1 × PBS resulted in ~ 70% high molecular weight species, suggesting that the intermolecular interactions were not solely due to disulfide bonding (Fig. 4D). Thus, if TL1A could form transient non-covalent interactions which favored formation of intermolecular disulfide bonds, then these interactions could be inhibited by changing buffer conditions. We found that redox in a buffer consisting of only 20 mM sodium phosphate, pH 6.8 (without additional NaCl) resulted in ~ 90% stable dimeric species which retained its stability and displayed identical binding properties as the SEC-purified material suggesting that hydrophobic non-covalent intermolecular interactions also contributed to transient aggregates that could be stabilized by intermolecular disulfide bonds during secretion (Fig. 4E).

Although the trimeric interface was thought to represent a tight binding site for monomer subunits of the TL1A trimer, individual TL1A subunits in the scTL1A format could have paired in *trans* with subunits from another TL1A molecule or with subunits from the TL1A molecule on the adjacent Fc subunit. To address the latter question, we determined a low-resolution structural model of Fc-scTL1A by solution x-ray scattering (Fig. 4F, Supplementary Table S1, Supplementary Fig. S3). The reconstructed molecular envelope suggests that the two TL1A moieties are splayed away from one another in the structure and that pairing across subunits was unlikely. Additionally, the consistency in scattering curves across three concentrations (3, 6, and 12 mg/mL) suggested the molecule did not undergo concentration-dependent aggregation.

### A TL1A variant maintained binding to DR3 but not to DcR3

**Figure 5:**
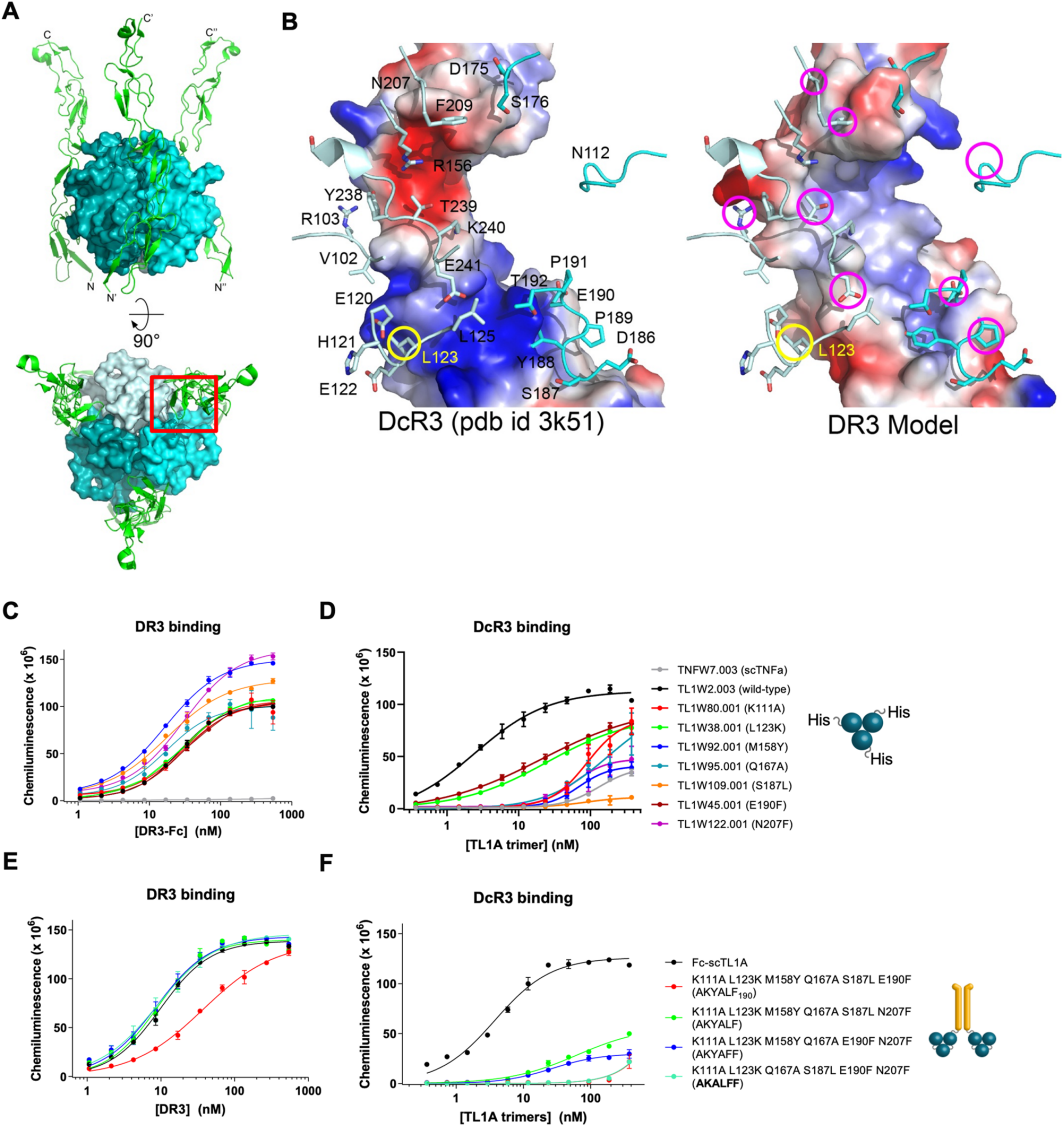
TL1A Variants Specifically Maintained Binding to DR3 but not DcR3. (**A**) Cartoon representation of the complex of DcR3 bound to TL1A, based on PDB ID 3K51. DcR3 molecules are shown as green ribbons, and TL1A subunits are shown as blue surfaces. The binding surface on TL1A for DcR3, at the interface between adjacent monomers, is boxed in red. The boxed area is shown in B. (**B**) Illustration of the residues on TL1A that interact with DcR3 (left) based on PDB ID 3K51 or with DR3 (right) based on a structural model of the TL1A:DR3 complex. Residues mutated in this study are circled in magenta or yellow (L123). (**C**–**F**) ELISA analysis of the abilities of single point mutants of TL1A (**C**, **D**) or combination mutants (**E**, **F**) to bind DR3 (**C**, **E**) or DcR3 (**D**, **F**). Mutations are indicated in the Figure. Structural images were generated using Pymol (Schrödinger): https://pymol.org/2/.

TL1A-mediated co-stimulation of T cells can be dampened in vivo by circulating DcR3. We sought to generate a variant of TL1A which would retain binding to DR3 but which would not be bound up by DcR3. An important study focused on mutations in TL1A that could modulate its interactions with DcR3^13^. In particular, mutation of E120A, E122A, L123A, G124D, Y188F, K240A, E241A, D242A, and K243A could disrupt interaction with DcR3, although the effect of these mutations on DR3 binding was not reported^13^. Although the cysteine-rich domains of DR3 and DcR3 are structurally similar (Fig. 5A,B), they share only 26% sequence identity, presenting an opportunity for differential binding by a TL1A variant. We used the crystal structure of TL1A bound to DcR3^14^ and models of TL1A bound to DR3 to guide mutagenesis (Fig. 5B). We noted that several residues in TL1A appeared to make significantly different contacts with DcR3 compared to DR3 and these were chosen for mutagenesis. The identities of the mutations were chosen to optimize the side-chain interactions between TL1A and DR3 and to simultaneously disrupt interactions with DcR3. Of the 83 variants tested in His-TL1A format, several retained or enhanced binding to DR3 and disrupted binding to DcR3 (Fig. 5C,D, Table 3, Supplementary Fig. S3A and B, Supplementary Table S2). Our binding data are largely consistent with those found previously by Zhan et al. In particular, mutation of L123, Y188, or E241 could significantly disrupt binding to DcR3 in both studies, although we identified several unique sites for introducing differential binding.

**Table 3:** ELISA results for TL1A variants.

Molecule	EC_50_ (nM): DR3	Rel EC_50_ DR3	EC_50_ (nM): DcR3	Rel EC_50_ DcR3
TL1W2	26.2 ± 1.9	1.0	4.6 ± 0.9	1.0
Fc-scTL1A (TL1W14)	9.7 ± 1.0	1.0	6.5 ± 0.9	
TNFγ	No binding	NA	No binding	NA
TL1W80 (K111A)	30.0 ± 12.2	1.1	89.8 ± 1.1	
TL1W38 (L123K)	29.4 ± 4.1	1.1	24.7 ± 1.4	
TL1W92 (M158Y)	16.6 ± 2.9	0.6	69.2 ± 1.8	
TL1W95 (Q167A)	17.1 ± 11.5	0.7	175.9 ± 2.3	
TL1W109 (S187L)	17.7 ± 6.3	0.7	85.5 ± 1.9	
TL1W45 (E190F)	31.4 ± 4.3	1.2	21.2 ± 1.3	
TL1W122 (N207F)	30.2 ± 5.8	1.2	58.5 ± 1.8	
TL1W328.001 (K111A L123K M158Y Q167A S187L N207F)	8.8 ± 2.8	0.8	No binding	NA
TL1W329.001 (K111A L123K M158Y Q167A E190F N207F)	8.7 ± 2.6	0.9	No binding	NA
TL1W331.001 (K111A L123K Q167A S187L E190F N207F)	9.0 ± 2.7	0.9	No binding	NA
TL1W327.001 (K111A L123K M158Y Q167A S187L E190F)	37.5 ± 3.6	3.9	No binding	NA

We selected seven single point mutations to generate combination mutants in the Fc-scTL1A format and screened them for binding to DR3 or DcR3 by ELISA (Fig. 5E,F, respectively). Except for the K111A, L123K, M158Y, Q167A, S187L, E190F variant (AKYALF_190_) which showed modestly decreased DR3 binding, the other 3 combination mutants maintained similar binding to DR3 compared to wild-type TL1A (Fig. 5E), but failed to bind DcR3 significantly (Fig. 5F, Supplementary Fig. S4). The Fc-scTL1A-AKALFF variant, harboring the K111A, L123K, Q167A, S187L, E190F, N207F mutations, was favored based on its binding selectivity to DR3 but not to DcR3.

**Figure 6:**
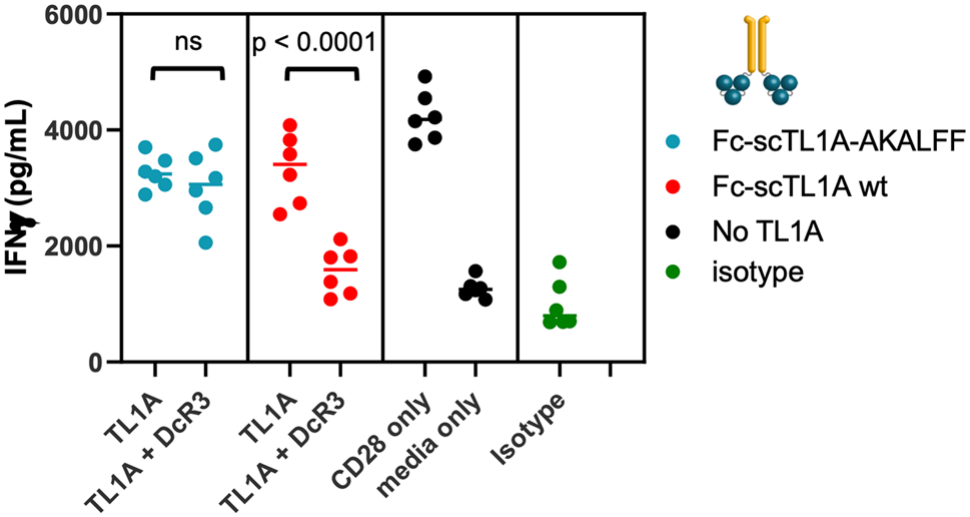
Fc-TL1A-AKALFF was Immune to DcR3-Based Inhibition. T cells were treated with 0.1 mg of immobilized anti-CD3 antibody alone or in the presence of exogenous DcR3 at 10 mg/mL. Either Fc-scTL1A (red) or Fc-scTL1A AKALFF (blue) was used to co-stimulate T cells. Anti-CD28 Ab was used as a co-stimulatory positive control. Anti-CD3-activated T cells without co-stimulatory signal was used as negative control. IFNg levels were measured by MSD assay. Stastical significance was determined using an unpaired T-test for each construct and data represents averages from three independent experiments using a single T cell donor. Graphs were generated using Graphpad Prism (Version 9): https://www.graphpad.com/scientific-software/prism/.

We then determined whether the Fc-scTL1A-AKALFF variant could retain the ability to co-stimulate T cells and would be resistant to DcR3-mediated competition (Fig. 6). Indeed, Fc-scTL1A-AKALFF was able to co-stimulate anti-CD3-activated T cells at levels similar to wildtype TL1A (Fig. 6). The addition of exogenous DcR3 inhibited T cell co-stimulation by wildtype Fc-scTL1A, but not by Fc-scTL1A-AKALFF. This is consistent with binding data showing AKALFF mutations abolished binding to DcR3 without affecting binding to DR3. Together, these findings suggest that the Fc-scTL1A-AKALFF variant designed here possessed properties suitable for both functional activity and therapeutic development.

## Discussion

Many solid tumors create an immune-suppressive environment in which tumor-infiltrating lymphocytes (TILs) are prevented from acting against tumor cells^17^. The successes of immune-checkpoint blockade therapies targeting PD-L1 and CTLA-4 suggest the potential of additional targets to overcome tumor immune suppression. Clinical targeting of TNFSF ligands and their receptors to combat cancers has shown promise, with a number of antibodies and TNFSF ligand molecules approved or in clinical development^1^. TL1A can co-stimulate T cells through the engagement of DR3 on T cells, allowing enhanced activation against tumor cells. However, agonizing DR3 using therapeutic ligands could be rendered ineffective due to up-regulation of soluble DcR3 which blocks DR3 binding and thus dampens TL1A activity.

Here, we designed an Fc-scTL1A ligand with high monodispersity and stability which maintains binding to DR3 while showing no measurable binding to DcR3. One challenge for the development of therapeutic ligands is their relatively short serum half-life (hours) compared to antibodies (~ 3 weeks). Fusion to either an Fc or serum albumin, both of which display long serum half-lives due to their abilities to undergo FcRn-mediated recycling have been successful methods to extend the half-life of protein therapeutics^15^. We tested several architectures of TL1A ligand, including single-chain versions of the trimeric ligand, in which each domain was linked by a Gly-Ser linker, as well as both human serum albumin- and Fc-fusions. A version of TL1A in which the ligand was formatted as a single-chain and was fused onto the C-terminus of the Fc showed the highest monodispersity upon purification. Surprisingly, molecules in which TL1A was fused onto the N-terminus of the Fc failed to show binding to receptors, although the mechanism preventing binding was unknown. TL1A contains two cysteines (C162, C202) which form an intra-molecular disulfide bond and stabilize a loop which is critical for binding DR3 (Fig. 1A,B). Recombinant production of TL1A resulted in ~ 20% aggregate, and this aggregate was largely mediated by improper inter-molecular disulfide bond formation, primarily mediated by C162. In this study, mutation of either C162 or C202 to A/S/L resulted in total disruption of DR3 binding. Zhan et al. reported that these cysteines could be mutated without disrupting binding to DcR3, which is consistent with our findings^13^. To overcome this production challenge, we showed that redox in low salt conditions favored re-formation of the appropriate intra-subunit disulfide bonds and resulted in lower amounts of aggregates. Non-antibody biologics often display heterogeneity of glycosylation which presents a challenge for characterization of clinical batches. The TL1A sequence contains two N-linked glycosylation motifs starting at positions 133 and 229. Mutation of N133Q but not N229Q could maintain binding affinity with no apparent changes in solubility or expression (Supplementary Fig. S4C), representing a potential improvement for therapeutic development. The Fc-scTL1A molecule displayed the ability to co-stimulate T cells in vitro (Figs. 2E,F, 3), Although Fc-TL1A and Fc-scTL1A displayed similar functional activity, Fc-scTL1A had more favorable monodispersity. In these studies, the Fc retained the ability to engage Fcg receptors, and in vivo, clustering of the Fc on immune cells via these receptors may contribute to additional activity. However, in vitro experiments where Fcg receptor-expressing cells were absent suggested that the TL1A ligand was capable of co-stimulating T cells in the absence of additional clustering. Co-stimulatory ligands featuring an active Fc may mediate effector functions, such as antibody-dependent cellular cytotoxicity, against T cells, which is undesirable in a therapeutic setting, and thus an Fc in which binding to Fcg receptors is ablated may be desirable^18^.

We designed mutations in TL1A based on the crystal structure of TL1A bound to DcR3 and based on modelling of the interaction between TL1A and DR3. Previous studies by Zhan et al. suggested that a TL1A variant could be developed which specifically interacts with DR3^13^. Our studies suggested that mutations in TL1A which disrupt multiple interactions with DcR3 were needed to achieve full abrogation of binding (Fig. 4C–F, Supplementary Fig. S4A, Table 3, Supplementary Table S2). Of these, seven mutants were selected for further analysis and were combined and tested in the Fc-scTL1A architecture, showing consistent binding results (Fig. 5E,F). Favorable mutants were characterized as having normal or enhanced binding to DR3 (relative EC50 < 1) and weaker binding to DcR3 (relative EC50 > 1). This suggested that the individual modifications to enhance the stability, monodispersity, and specificity of TL1A could be combined to generate a therapeutically viable molecule. Indeed, Fc-TL1A K111A, L123K, Q167A, S187L, E190F, N207F (FcTL1A-AKALFF) displayed identical binding to DR3 (compared to wild-type TL1A) but displayed no significant binding to DcR3. This binding specificity was recapitulated in the T cell activation assay, where addition of exogenous DR3 could inhibit T cell activation by Fc-scTL1A or Fc-scTL1A-AKALFF but DcR3 could inhibit only Fc-scTL1A (Fig. 6).

A particular challenge for TNFSF-targeting therapeutics is tumor targeting. Systemic T cell activation is associated with the potential for rapid and severe toxicity associated with cytokine release via off-tumor T cell activation and the potential for reactivation-induced T cell death, wherein tumor-infiltrating T cells (TILS) undergo apoptosis in response to over-activation by bispecific T cell engagers (bsTCE;^19^. To overcome this, co-stimulatory molecules (antibodies or ligands) have been generated as bispecific antibodies which can bind both co-stimulatory receptors and to a tumor antigen. One study showed that an anti-EGFR × LIGHT-Fc BsAb could target tumors and activate LTβR signaling to recruit TILS and showed anti-tumor efficacy^20^. Results from this work suggest that targeting co-stimulatory activity to a tumor has therapeutic promise. DcR3 is upregulated in a number of solid tumor types, particularly colon, pancreatic, and stomach cancers^21^, suggesting that DcR3 in these tumors may be a mechanism of T cell inhibition and that T cells within these tumors could be stimulated by a TL1A ligand which can overcome the DcR3-mediated sink. Bispecific antibodies which can deliver the Fc-scTL1A-AKALFF specifically to these tumors may offer similar anti-tumor activity with limited risk of systemic T cell activation. The Fc-scTL1A-AKALFF variant developed in this work was designed to function as a module which can be joined with a tumor-targeting moiety to specifically activate T cells in the tumor micro-environment, and its favorable monodispersity, stability, and activity suggest it will mediate anti-tumor activity.

## Methods

### Proteins

TL1A constructs and variants were optimized for human codon usage and cloned into a mammalian expression vector. Constructs were transfected into Expi293 cells (Thermo) and expressed according to the manufacturer’s protocol. Proteins were purified by either Ni–NTA affinity chromatography (His-TL1A and HSA-TL1A) or by mAbSelect SuRe (GE Healthcare Life Sciences) (Fc-TL1A) according to the manufacturer’s protocol. Preparative gel-filtration was performed using a Sepax SRT-C SEC-300 column (Sepax Technologies, Inc.). Gel-filtration was performed in buffer consisting of 20 mM sodium phosphate, pH 6.8.

### SEC-MALS

SEC separations were performed on a Waters Acquity UPLC system consisting of an H-class quaternary pump, FTN autosampler, heated column compartment and TUV detector. Samples were maintained at 4 °C in an autosampler until analysis. Aliquots (5 µg) of the samples were analyzed on a Waters BEH SEC column (200 A, 1.7 µ dp, 4.6 × 150 mm) maintained at 25 °C. The mobile phase was PBS, pH 7.4 at a flow rate of 300 µL/min, and UV absorbance was monitored at 280 nm. The entire flow from the TUV detector was directed to a Wyatt µDAWN detector equipped with additional DLS channel at a scattering angle of 135° and a serially connected Wyatt Optilab T-rEX refractive index detector. The LC was operated under control of MassLynx V4.2 SCN1007 software, while Wyatt components were managed with Astra V7.3.2. UV data was acquired by Astra via auxiliary input of 0.2 AU/V signal. All data was processed with Astra V7.3.2.

### MSD electrochemiluminescence cytokine assay

U-PLEX TH1 TH2 cytokine (cat # K15010B-2) plates were coated with 25 ml of cell culture supernatant overnight at 4 °C. The plates were washed 3X with PBS with 0.05% Tween 20 (PBST). Anti-human Fc detection reagent at 2 mg/ml final concentration was applied for 1 h at RT with shaking. The plates were washed 3X with PBST. 150 ml of 2X read buffer was added to the plates, and data was collected on an MSD instrument. Raw data was processed in MSD Discovery Workbench and imported into GraphPad Prism 8 software. Data were analyzed and plotted based on the results of three independent experiments, and statistics were generated using GraphPad Prism 8.

### ELISA

All ELISA-based measurements were collected in triplicate, and error values report the standard error among measurements. Recombinant DR3-Fc (R&D Systems, cat. # 943-D3, dimeric MW ~ 92 kDa) was non-specifically immobilized onto 96-well White Maxisorp plates (Nunc, cat. # 436110) through dilution to 10 mg/mL (overnight, 4 °C). Plates were blocked for 1 h at room temperature with Casein buffer, 250 ml/well. TL1A was added at the indicated concentrations at 50 mL/well diluted in StartingBlock PBS, at 25 mg/mL*, diluting 3 × over each well on a 96-well White Maxisorp plate (Nunc, cat#: 436110) and incubated overnight at 4 °C. Note that TL1A variant concentrations were normalized to moles of TL1A trimer/molecule. For example, 1 molecule of His-TL1A contains 1 TL1A trimer, and thus 25 mg/mL of TL1W2 contains 376 nM TL1A trimers. Conversely, TL1W14 contains 2 TL1A trimers per molecule, and has a different molecular weight. Thus, 25 mg/mL TL1W14 = 290 nM. Therefore, 376 nM is equivalent to 32.4 mg/mL. Plates were then washed 3 × with TBST. Bound TL1A was detected by addition of 100 µl/well polyclonal rabbit anti-human TL1A-biotin antibody, diluted 1:1,000 in Starting Block PBS and incubated for 1 h at room temperature with shaking ~ 150 rpm. Anti-TL1A was detected by addition of 100 ml/well streptavidin-HRP conjugate, diluted 1:10,000 in Starting Block PBS and incubated for 1 h at room temperature with shaking ~ 150 rpm. Plates were washed 3 × with TBST. Detection was carried out by addition of 100 µl/well POD Chemiluminescence substrate (Roche, cat#: 11582950001) immediately prior to reading plates. Luminescence was detected using a Wallac Envision plate reader.

### In vitro T cell activation

96-well U-bottom tissue culture plates (Midwest Scientific, cat. # TP92097) were coated with 0.1 or 0.01 mg/mL of anti-CD3 (BioLegend, cat. # 317304, clone OKT3) in PBS overnight at 4 °C. Plates were then washed three times with complete RPMI media (RPMI + 10% FBS). Pan T cells were isolated from peripheral blood mononuclear cells (PBMCs) using negative selection (Miltenyi Biotec, cat. #130-096-535). 30,000 pan T cells were plated in each well of anti-CD3 pre-coated 96-well U-bottom plates. Engineered TL1A ligands and/or 0.1 mg/mL of anti-CD28 (BioLegend, cat. # 302,914) were added to appropriate wells. To assess DcR3-mediated inhibition on T cell activation, recombinant DcR3 protein (R&D cat# 142-DC-100) was added to a final concentration of 30 nM to anti-CD3 stimulated T cells with or without TL1A ligands.

### In vivo T cell co-stimulation

TL1A constructs were evaluated for the ability to co-stimulate T cells to produce cytokine in an in vivo mouse model. Suboptimal dose of anti-mouse CD3 antibody (2 mg/mouse) (BioXCell, clone 145-2C11 F(ab)2 fragment; cat. # BE0001-1FAB) was injected intravenously into C57BL/6 mice. Fc-TL1A W3 or Fc-scTL1A W14 (30 µg per mouse) was injected intraperitoneally. Fc-TL1A W3 (100 µg per mouse) was injected intraperitoneally into mice in the absence of anti-mouse CD3 stimulation. Mice were bled 6 h later. Sera were collected and frozen. Frozen sera were thawed and diluted 1:10 for mouse IFNg ELISA (Invitrogen, cat. # 88-8314-86). Briefly, Nunc MaxiSorp 96-well plates were pre-coated with capture antibody overnight at 4 °C. Plates were washed and then blocked with ELISA diluent for 1 h at room temperature. Plates were washed with Wash Buffer. Diluted samples were loaded into plates and incubated for 2 h at room temperature. Plates were washed with Wash Buffer 3 to 5 times. Diluted detection antibody was added to each well, incubated for 1 h at room temperature, and washed. Diluted Streptavidin-HRP detection reagent was added, incubated for 30 min at room temperature, and washed. TMB substrate solution was added and incubated at room temperature for 15 min. Stop solution was added to each well. Plates were read in a SpectraMax ELISA plate reader.

### Modeling of the TL1A-DR3 interaction

Models of the TL1A-DR3 complex were generated using the protein modeling component of MOE (2016.08; Chemical Computing Group, Inc., Montreal, QC, Canada). The sequence of DR3 (Uniprot ID O95407) and the structure of TL1A bound to DcR3 (PDB ID 3K51) were used as inputs^14^.

### Solution X-ray scattering

Scattering data collection was carried out on an in-house x-ray source at 20 °C. Fc-scTL1A samples were analyzed in 20 mM HEPES HCl pH 6.5, 100 mM NaCl. To limit radiation damage, the samples were continuously oscillated inside the cuvette during the exposures. Independent measurements were collected from each sample at different concentrations (3, 6, or 12 mg/mL), checking the linear dependence of the scattering intensity at zero angle as a function of concentration to ensure lack of aggregation (Supplementary Table S1). Data normalization, solvent subtraction and Guinier analysis were done using the BioXTAS RAW software. Data analysis was carried out using the ATSAS software suite, including the programs GNOM^22^, used to calculate the distance distribution function P(*r*), and DAMMIF^23^, used in automated bead modelling for shape determination. To generate the models of Fc-scTL1A, 2-fold symmetry constraints were imposed, which produces more reliable envelopes by reducing noise^24^. For each sample, 5 independent shape models calculated with DAMMIF were averaged using DAMAVER to produce the final ab initio envelopes. Surface rendering was done with Chimera^25^.

### Surface Plasmon resonance

Goat anti-human-Fc was immobilized at 30 mg/ml, Acetate, pH 5.0 on L1-L6. DR3, DcR3 were immobilized at 5 or 1 mg/mL. The desired absolute ligand levels for either indirect capture format or immobilization formats are the range that can produce final analyte binding signals between 50~200 RUs. BSA was used as a non-binding analyte control. Experiments were performed at 25 °C, with PBST (1X DPBS, 0.005% (w/v) Tween) as the running buffer. A k_off_ screen for each of the 14 TL1A variants was performed first at 1 mM to evaluate the optimal capture level, analyte concentration and dissociation time needed. Each of the variants was analyzed at 0.1 mM in threefold dilution series over DcR3 captured 4 different ligand densities. Raw data were processed and analysed in ProteOn Manager software (BioRad, version 3.1.0.6). Kinetic analysis was done by grouping the k_on_, k_off_ and RUmax, and holding the RI constant and equal to zero. The Chi2 value was used to assess the quality of the fit. Data were evaluated only qualitatively.

## Date availability

The datasets used and/or analysed during the current study available from the corresponding author on reasonable request.

## Supplementary Information

Supplementary Information.
