# Supplementary material for: A stable, engineered TL1A ligand co-stimulates T cells via specific binding to DR3
Source: Sci Rep. 2022 Nov 29;12:20538. doi: 10.1038/s41598-022-24984-y (PMC9709071; doi:10.1038/s41598-022-24984-y)
Supplement: Supplementary file 1 — Supplementary Information. [file 41598_2022_24984_MOESM1_ESM.docx]

**A Stable, Engineered TL1A Ligand Co-stimulates T cells via Specific Binding to DR3**

Adam Zwolak^1^* ^ǂ^, Szeman Ruby Chan^2 ǂ^, Paul Harvilla^1^, Sally Mahady^2^, Anthony A. Armstrong^1^, Leopoldo Luistro^2^, Ninkka Tamot^1^, Douglas Yamada^2^, Mehabaw Derebe^4^, Steven Pomerantz^1^, Mark Chiu^5^, Rajkumar Ganesan^6^, Partha Chowdhury^7^

^1^Biologics Discovery, Janssen Research & Development, LLC, Spring House, PA, 19477, USA.

^2^Oncology Discovery, Janssen Research & Development, LLC, Spring House, PA, 19477, USA.

^4^Merck Research Laboratories, Discovery Biologics, Protein Sciences, South San Francisco, CA, USA

^5^Tavotek Biotherapeutics, Spring House, PA, USA.

^6^Immunotherapeutics, Amgen, South San Francisco, CA, USA.

^7^Cell Engineering and Early Development, Janssen Research & Development, Spring House, PA, USA

^ǂ^These authors contributed equally

*Correspondence: [azwolak1@its.jnj.com](mailto:azwolak1@its.jnj.com)

**SUPPORTING INFORMATION**

**Supplementary Method**

SEC separations were performed on a Waters Acquity UPLC system consisting of an H-class quaternary pump, FTN autosampler, heated column compartment and TUV detector.  Samples were maintained at 4 °C in autosampler until analysis.   Aliquots (5 µg) of the samples were analyzed on a Waters BEH SEC column (200 A, 1.7 µ dp, 4.6 x 150 mm) maintained at 25°C. The mobile phase was PBS, pH 7.4 at a flow rate of 300 µL/min, and UV absorbance was monitored at 280 nm.  The entire flow from the TUV detector was directed to a Wyatt µDAWN detector equipped with additional DLS channel at a scattering angle of 135° and a serially connected Wyatt Optilab T-rEX refractive index detector.  The LC was operated under control of MassLynx V4.2 SCN1007 software, while Wyatt components were managed with Astra V7.3.2.  UV data was acquired by Astra via auxiliary input of 0.2 AU/V signal.  All data was processed with Astra V7.3.2

| **Supplementary Table S1.** Solution x-ray scattering analysis | | | | |
| --- | --- | --- | --- | --- |
| Concentration (mg/mL) | 0 (extrapolated) | 2.97 | 5.93 | 11.85 |
| I(0) / concentration | 1.55 | 1.5 | 1.53 | 1.55 |
| Guinier Points | 1 | 3 | 3 | 3 |
|  | 9 | 13 | 12 | 12 |
| Quality | 99% | 86% | 90% | 91% |
| R_g_ (Å; Guinier) | 48.6 | 48.7 | 49.4 | 48.9 |
| R_g_ (Å; P(r)) | 49.8 | 49.4 | 49.4 | 48.5 |
| D_max_ (Å) | 166 | 163 | 166 | 160 |
| V_Porod_ (Å^3^) | 341.8 | 331.4 | 332.2 | 320.9 |
| V_DAM_ (Å^3^) | 387.6 | 375.9 | 383.8 | 372.8 |
| MW_Porod_ (kDa) | 213.6 | 207.1 | 207.6 | 200.6 |

| **Supplementary Table S2.** Binding comparison of other mutants | | | | |
| --- | --- | --- | --- | --- |
| **Molecule** | **EC_50_ (nM):**  **DR3** | **Rel EC_50_ (nM)**  **DR3** | **EC_50_ (nM):**  **DcR3** | **Rel EC_50_**  **DcR3** |
| Fc-scTL1A | 26.2 ± 1.9 | 1.0 | 6.5 ± 0.9 | 1.0 |
| TL1W2 (wt) | 26.2 | 1.0 | 4.6 | 1.0 |
| TL1W33 (R103A) | 25 | 1.0 | 3.3 | 0.7 |
| TL1W78 (R103H) | 77.2 | 2.9 | 8.1 | 1.8 |
| TL1W31 (R103Q) | 9.5 | 0.4 | 2.5 | 0.5 |
| TL1W79 (R103E) | 203.7 | 7.8 | 7.1 | 1.5 |
| TL1W80 (K111A) | 30 | 1.1 | 16.3 | 3.5 |
| TL1W81 (K111S) | 34.4 | 1.3 | 50.9 | 11.1 |
| TL1W82 (K111E) | 25.8 | 1.0 | 10.6 | 2.3 |
| TL1W30 (N112E) | 16.4 | 0.6 | 4.7 | 1.0 |
| TL1W42 (F114A) | 77.7 | 3.0 | 11.4 | 2.5 |
| TL1W41 (E120A) | 100.5 | 3.8 | 32.6 | 7.1 |
| TL1W83 (E120K) | NA |  | 2.2 | 0.5 |
| TL1W84 (E120H) | 182.3 | 7.0 | 55.7 | 12.1 |
| TL1W39 (L123G) | 184.3 | 7.0 | 38.2 | 8.3 |
| TL1W40 (L123S) | 30.7 | 1.2 | 16.4 | 3.6 |
| TL1W37 (L123E) | 422.9 | 16.1 | 24.3 | 5.3 |
| TL1W38 (L123K) | 29.5 | 1.1 | 24.7 | 5.4 |
| TL1W85 (G124S) | 295.8 | 11.3 | 57.1 | 12.4 |
| TL1W86 (G124K) | NA |  | 2.9 | 0.6 |
| TL1W87 (G124D) | 14.8 | 0.6 | NA | NA |
| TL1W88 (R156A) | NA |  | 13.5 | 2.9 |
| TL1W89 (R156Y) | NA |  | 8.0 | 1.7 |
| TL1W90 (R156K) | 187.6 | 7.2 | 11.0 | 2.4 |
| TL1W91 (R156E) | NA |  | 8.3 | 1.8 |
| TL1W92 (M158Y) | 16.6 | 0.6 | 12.6 | 2.7 |
| TL1W93 (M158K) | 22.5 | 0.9 | 55.9 | 12.1 |
| TL1W94 (M158E) | 58.7 | 2.2 | 18.7 | 4.1 |
| TL1W95 (Q167A) | 17.1 | 0.7 | 32.0 | 7.0 |
| TL1W96 (R170E) | 87 | 3.3 | 16.4 | 3.6 |
| TL1W97 (K173S) | 131.3 | 5.0 | 6.7 | 1.5 |
| TL1W98 (K173R) | 2054 | 78.4 | 27.8 | 6.0 |
| TL1W99 (S176A) | 34.8 | 1.3 | 11.5 | 2.5 |
| TL1W100 (S176L) | 323.9 | 12.4 | 15.0 | 3.3 |
| TL1W101 (S176N) | NA |  | 20.4 | 4.4 |
| TL1W102 (S176K) | 181.5 | 6.9 | 29.2 | 6.3 |
| TL1W103 (T185A) | 57.9 | 2.2 | 11.5 | 2.5 |
| TL1W104 (T185L) | 49.4 | 1.9 | 19.3 | 4.2 |
| TL1W105 (T185N) | 179.5 | 6.9 | 11.3 | 2.4 |
| TL1W106 (T185D) | 123.5 | 4.7 | 79.9 | 17.4 |
| TL1W107 (D186Y) | 63.8 | 2.4 | 6.2 | 1.4 |
| TL1W108 (S187A) | 39.1 | 1.5 | NA | NA |
| TL1W109 (S187L) | 17.7 | 0.7 | 15.5 | 3.4 |
| TL1W110 (S187K) | 33.7 | 1.3 | 15.8 | 3.4 |
| TL1W111 (S187D) | 44.2 | 1.7 | 16.7 | 3.6 |
| TL1W32 (Y188A) | 565.1 | 21.6 | 7.6 | 1.7 |
| TL1W43 (Y188S) | 206.6 | 7.9 | 72.2 | 15.7 |
| TL1W29 (P189A) | 710.1 | 27.1 | 7.6 | 1.7 |
| TL1W44 (P189K) | 167.6 | 6.4 | 59.1 | 12.8 |
| TL1W112 (P189F) | 202.4 | 7.7 | 66.1 | 14.4 |
| TL1W113 (P189S) | 1002 | 38.2 | 19.8 | 4.3 |
| TL1W28 (E190G) | 22.6 | 0.9 | 5.7 | 1.2 |
| TL1W45 (E190F) | 31.4 | 1.2 | NB |  |
| TL1W114 (T192A) | 23.3 | 0.9 | 10.0 | 2.2 |
| TL1W115 (T192F) | 14.2 | 0.5 | 10.6 | 2.3 |
| TL1W116 (T192K) | 77 | 2.9 | 23.3 | 5.1 |
| TL1W117 (T192E) | 112.9 | 4.3 | 12.0 | 2.6 |
| TL1W118 (S206A) | 41.7 | 1.6 | 8.8 | 1.9 |
| TL1W119 (S206F) | 87.5 | 3.3 | 8.1 | 1.8 |
| TL1W120 (S206K) | 114.4 | 4.4 | 91.0 | 19.8 |
| TL1W121 (S206E) | 105.3 | 4.0 | 17.2 | 3.7 |
| TL1W25 (N207A) | 13.4 | 0.5 | 3.7 | 0.8 |
| TL1W122 (N207F) | 30.2 | 1.2 | 10.6 | 2.3 |
| TL1W123 (N207S) | 26.8 | 1.0 | 8.6 | 1.9 |
| TL1W124 (N207k) | 110.1 | 4.2 | 15.4 | 3.4 |
| TL1W125 (N207E) | 506.1 | 19.3 | 9.8 | 2.1 |
| TL1W24 (F209A) | 280.6 | 10.7 | 30.7 | 6.7 |
| TL1W126 (F209W) | 56.8 | 2.2 | 13.7 | 3.0 |
| TL1W127 (Y238A) | 4624 | 176.5 | 8.9 | 1.9 |
| TL1W128 (Y238S) | NA |  | 10.5 | 2.3 |
| TL1W129 (Y238K) | 445.6 | 17.0 | 9.0 | 2.0 |
| TL1W130 (Y238R) | NA |  | 16.9 | 3.7 |
| TL1W131 (Y238E) | 300.3 | 11.5 | 6.6 | 1.4 |
| TL1W132 (T239A) | 144.4 | 5.5 | 27.2 | 5.9 |
| TL1W27 (T239E) |  | 0.0 | 34.3 | 7.5 |
| TL1W133 (T239F) | NA |  | 10.8 | 2.3 |
| TL1W134 (T239K) | NA |  | 21.5 | 4.7 |
| TL1W46 (T239W) | 283.7 | 10.8 | NA | NA |
| TL1W135 (K240A) | NA |  | 8.5 | 1.9 |
| TL1W136 (K240F) | 265.5 | 10.1 | 178.1 | 38.7 |
| TL1W137 (K240S) | 335.7 | 12.8 | 11.8 | 2.6 |
| TL1W138 (K240D) | NA |  | 59.5 | 12.9 |
| TL1W47 (E241A) | 259.9 | 9.9 | 1311.0 | 285.0 |
| TL1W33 (E241L) | NA |  | 5.7 | 1.2 |
| TL1W139 (E241Q) | NA |  | 17.3 | 3.8 |

***Rel EC50 DR3 = relative EC50 values (EC50 value / EC 50 value of TL1W2); *Rel EC50 DRc3 = relative EC50 values (EC50 value / EC 50 value of TL1W2)**

**
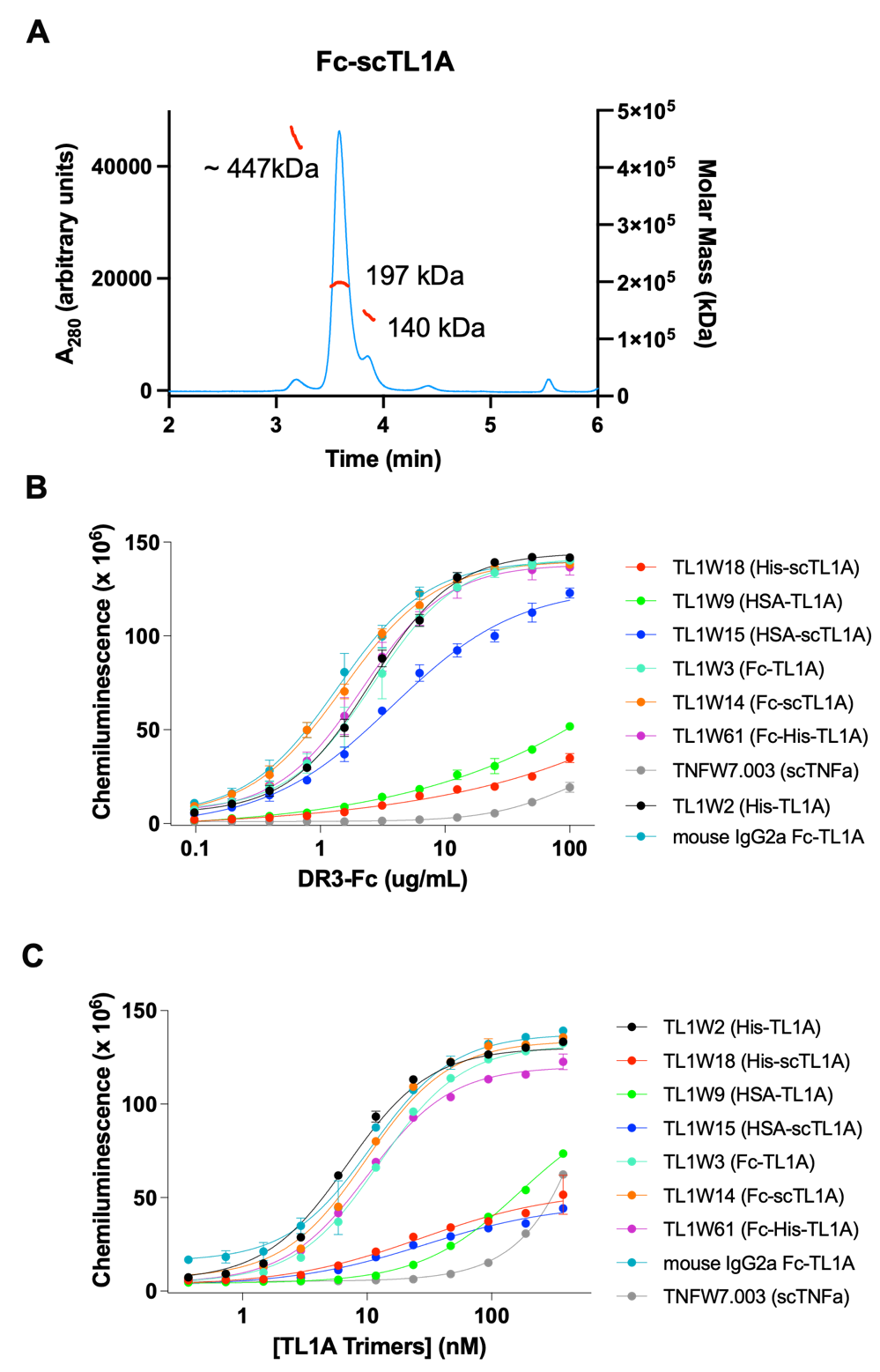
**

**Supplementary Figure S1. Comparison of ELISA formats for binding between TL1A constructs and DR3.** (A) SEC-MALS analysis of the oligomeric species of Fc-scTL1A. Absorbance at 280 nm is plotted on the left y-axis while molar mass is plotted on the right y-axis. Selected TL1A variants were analyzed for their abilities to bind DR3 when immobilized (B) and when titrated over immobilized DR3 (C). TL1A variants are indicated on the graphs. Graphs were generated using Graphpad Prism (Version 9): https://www.graphpad.com/scientific-software/prism/.


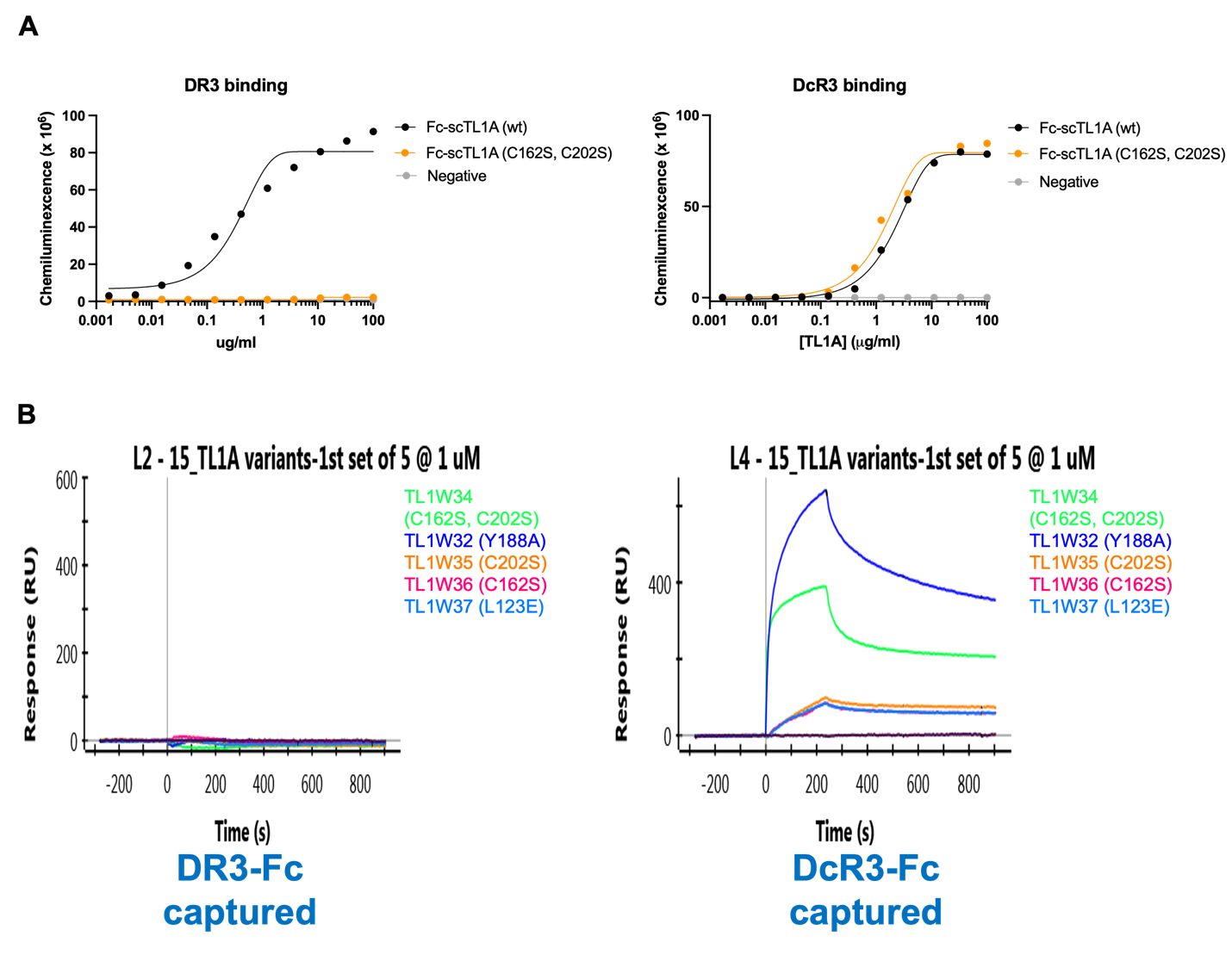


**Supplementary Figure S2. TL1A-C162S, C202S Can Bind DcR3 but not DR3.** Selected TL1A variants in His-TL1A format were analyzed for their abilities to bind DR3 (left) and DcR3 (right) by ELISA (A) and SPR (B). ELISA analyses were performed as in Figure 2. For SPR analyses, Fc-DR3 / DcR3 were immobilized using goat anti-human Fc, and TL1A variants were flowed over the immobilized receptor. Variants are indicated on the graph. Graphs were generated using Graphpad Prism (Version 9): https://www.graphpad.com/scientific-software/prism/.


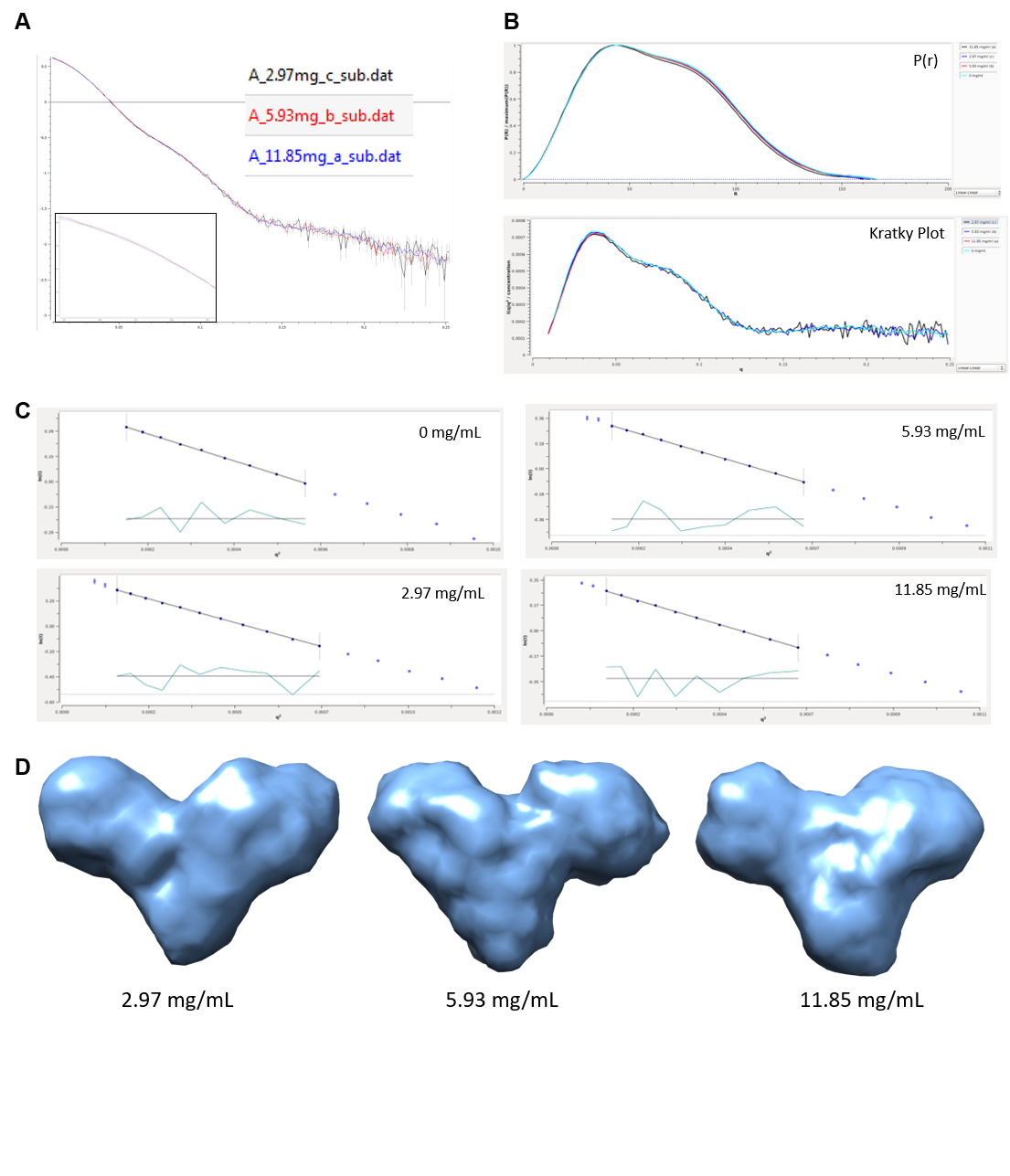


**Supplementary Figure S3.** **Solution X-ray Scattering Analysis of Fc-scTL1A** **Suggests TL1A Ligands Form Intra-subunit Trimers.** (A) Buffer subtracted scattering curves generated by AAP displayed in Primus and scaled to one another based on points 10-100. Point 1-170 shown. Inset shows a magnification of the same at the low q range. Curves indicate little aggregation of sample at 11.85 mg/mL. (B) Distance distribution (P(r)) and Kratky plots of scattering data. (C) Guinier analysis of scattering data. (D) *Ab initio* models of Fc-scTL1A from data collected at the indicated concentrations. Data were processed and graphs were generated using the ATSAS software suite (Version 3.1): <https://www.embl-hamburg.de/biosaxs/software.html>. Surface representations were generated using Pymol (Schrödinger): https://pymol.org/2/.


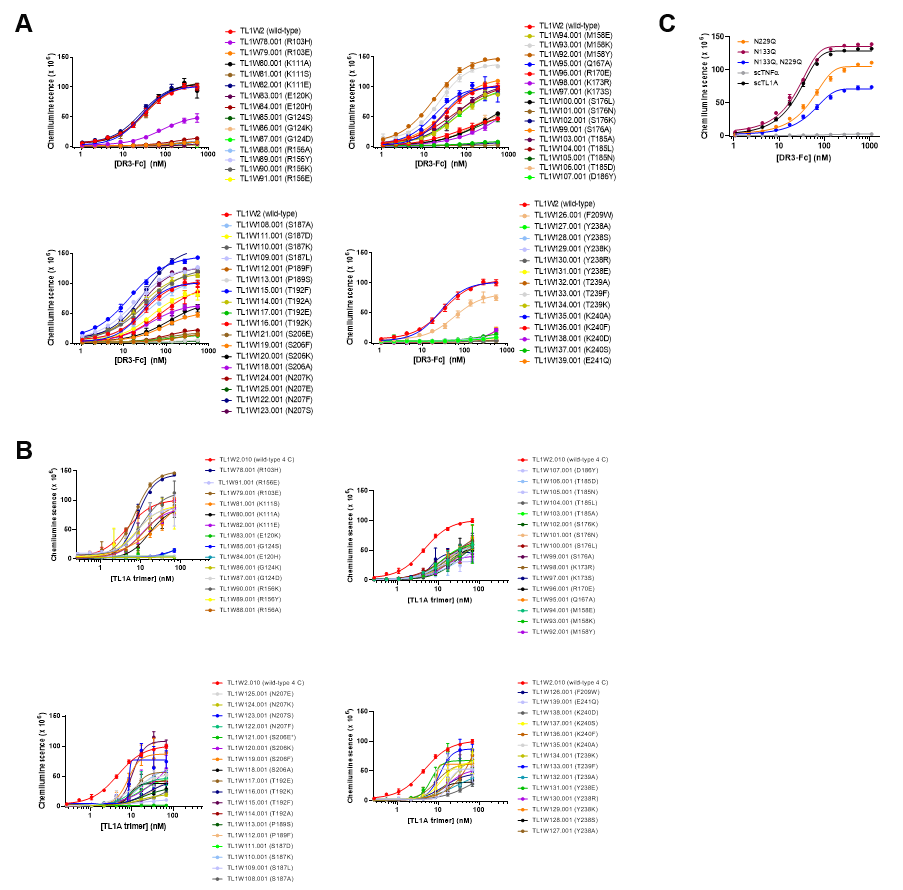


**Supplementary Figure S4.** **Additional TL1A Variants Could Modulate DR3 or DcR3 Binding.** (A) Single-point variants of TL1A designed to enhance specificity for DR3. Mutations are indicated on the graphs. (B) Mutants designed to assess whether glycosylation was involved in binding to DR3. Graphs were generated using Graphpad Prism (Version 9): https://www.graphpad.com/scientific-software/prism/.
